# Sustainability in radiation oncology: opportunities for enhancing patient care and reducing CO_2_ emissions in breast cancer radiotherapy at selected German centers

**DOI:** 10.1007/s00066-024-02303-w

**Published:** 2024-09-24

**Authors:** Ahmed Bedir, Maximilian Grohmann, Sebastian Schäfer, Matthias Mäurer, Steffen Weimann, Julian Roers, Dominik Hering, Michael Oertel, Daniel Medenwald, Christoph Straube

**Affiliations:** 1 Department of Radiation Oncology, Health Services Research Group, University Hospital Halle (Saale), Ernst-Grube-Str. 40, 06120 Halle (Saale), Germany; 2 Department of Radiotherapy and Radiation Oncology, University Medical Center Hamburg-Eppendorf, Martinistr. 52, 20246 Hamburg, Germany; 3 Department for Radiotherapy and Radiation Oncology, University Hospital Jena, Friedrich-Schiller-University, Am Klinikum 1, 07747 Jena, Germany; 4 Department of Radiation Oncology, University Hospital Muenster, Albert-Schweitzer-Campus 1 A1, 48149 Münster, Germany; 5 Department of Radiation Oncology, University Hospital Halle (Saale), Ernst-Grube-Str. 40, 06120 Halle (Saale), Germany; 6 Department of Radiation Oncology, Klinikum Landshut, Robert-Koch-Str. 1, 84034 Landshut, Germany

**Keywords:** Climate change, Health services, Carbon footprint, Fossil fuels, Radiation fractions, Travel distance

## Abstract

**Background and objective:**

Radiotherapy often entails a substantial travel burden for patients accessing radiation oncology centers. The total travel distance for such treatments is primarily influenced by two factors: fractionation schedules and the distances traveled. Specific data on these aspects are not well documented in Germany. This study aims to quantify the travel distances for routine breast cancer patients of five radiation oncology centers located in metropolitan, urban, and rural areas of Germany and to record the CO_2_ emissions resulting from travel.

**Methods:**

We analyzed the geographic data of breast cancer patients attending their radiotherapy treatments and calculated travelling distances using Google Maps. Carbon dioxide emissions were estimated assuming a standard 40-miles-per-gallon petrol car emitting 0.168 kg of CO_2_ per kilometer.

**Result:**

Addresses of 4198 breast cancer patients treated between 2018 and 2022 were analyzed. Our sample traveled an average of 37.2 km (minimum average: 14.2 km, maximum average: 58.3 km) for each radiation fraction. This yielded an estimated total of 6.2 kg of CO_2_ emissions per visit, resulting in 156.2 kg of CO_2_ emissions when assuming 25 visits (planning, treatment, and follow-up).

**Conclusion:**

Our study highlights the environmental consequences associated with patient commutes for external-beam radiotherapy, indicating that reducing the number of treatment fractions can notably decrease CO_2_ emissions. Despite certain assumptions such as the mode of transport and possible inaccuracies in patient addresses, optimizing fractionation schedules not only reduces travel requirements but also achieves greater CO_2_ reductions while keeping improved patient outcomes as the main focus.

## Introduction

Radiotherapy (RT) is a cornerstone of modern oncologic treatment, with high relevance for almost all solid malignancies and application in both curative as well as palliative settings [1, 2]. The treatment usually includes several visits to the radiation oncology center (ROC) to gain informed consent, to perform computed tomography (CT)-based treatment planning, for the treatment itself, and for follow-up. The scope of the treatment itself ranges from one visit for radiosurgery or palliative RT for bone metastases, to 42 visits for conventionally fractionated RT (CRT) in prostate cancer. The optimal number of fractions is continuously debated, with the average ranging from 18 to 21 fractions per course as a mean over all malignant indications [3]. Recent trials across various cancers, including breast and prostate cancer as well as palliative care, advocate for short regimens, a practice that is increasingly recognized in international guidelines [4–22].

Since 2012, hypofractionation has been an optional standard of care (SOC) for early breast cancer in Germany. In contrast, for node-positive breast cancer, the German interdisciplinary S3 guideline of 2021 still considers conventional fractionation as the SOC but acknowledges hypofractionation as a viable option in selected cases. The 2023 German Gynecological Oncology Group (AGO) guideline also considers conventional fractionation as the SOC, but hypofractionation is also supported as a valuable treatment option. Both the S3 and AGO guidelines also recommend use of targeted intraoperative radiotherapy (TARGIT-IORT) for suitable patients [23–25]. Given similar oncologic results with moderate hypofractionation and a better safety profile, moderate hypofractionation is likely to gain even broader acceptance in the future.

Besides medical, economical, and personal advantages associated with moderate hypofractionation in line with current guidelines, there are obvious ecologic advantages too. RT is associated with significant CO_2_ emissions that are both directly and indirectly linked to the numbers of fractions. Direct emissions were recently evaluated for proton- and photon-based ROCs, with mean CO_2_ emissions of 23 kg for proton and 0.7–4.1 kg for photon radiotherapy courses, with additional power consumption during idle and standby time as well as for quality assurance [26, 27]. Beyond these direct emissions, a large proportion of CO_2_ emissions are due to patient travel, which is directly attributable to the fractionation scheme [26, 28–30]. For Germany, there is no information about the average travel burden of patients who undergo radiotherapy [31]; however, this information forms the basis for an assumption of current CO_2_ emissions as well as of the anticipated saving potential.

This article places emphasis on evaluating real travel distances for patients undergoing treatment in German ROCs. By using real-word averaged travel distances from five ROCs in Germany as a basis for an informed model, we aim to explore the extent of the impact of different fractionation schemes. Understanding the relationship between patient travel distances and CO_2_ emissions is crucial for identifying potential strategies for reducing the CO_2_ footprint associated with radiotherapy. We chose breast cancer cases for this project as there is large variability in the fractionation options available and because breast cancer irradiation is offered by all oncologic ROCs in Germany.

## Methods

### Study design and data collection

**Fig. 1:**
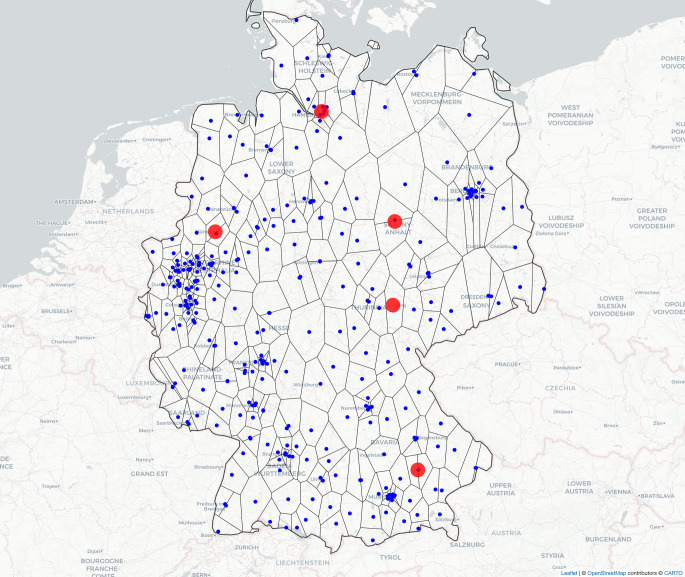
Voronoi diagram of radiation oncology centers (ROCs) in Germany. The *Blue dots* represent the ROCs in Germany while the *Red dots* represent the five participating ROCs. Due to the high density and close proximity of some ROCs, certain centers may overlap or appear partially obscured. *Red* centers have been superimposed onto *blue* ones to ensure visibility

This retrospective analysis utilized geographic data from 4405 breast cancer patients who underwent radiotherapy treatment during the 2018–2022 period at five German ROCs. The location of the participating ROCs and their relationship to nearby ROCs is shown using a Voronoi diagram (Fig. 1) created with the *deldir* package in R (R Foundation, Vienna, Austria; [32, 33]). A Voronoi diagram divides the map into regions, each centered around a specific ROC. Each region includes all areas that are closest to that ROC compared to any other. This helps to show which ROC is nearest for patients in different locations, illustrating the potential travel distances they face.

For each patient, the driving distance from their documented home address to the respective radiotherapy center was determined using the Google Maps *mapsapi* package in the R statistical program [34]. It was assumed that patients undergoing external-beam radiotherapy (EBRT) would need two additional journeys for radiotherapy planning and follow-up.

### Statistical analysis

Considering that the home addresses provided by the patients were not externally verified as well as the diverse geographic locations of the included centers (urban vs. rural), we used boxplot statistics to identify and exclude potential outliers (207 addresses; Table 1). Specifically, a data point is considered an outlier if it is located more than 1.5 times the interquartile range (IQR) away from the third quartile (Q3) upwards or from the first quartile (Q1) downwards. We then calculated the average distance traveled by the patients for each visit to their retrospective radiotherapy center along with the respective standard deviation.

**Table 1:** Outlier threshold and adjusted ranges for travel distances undertaken by breast cancer patients during 2018–2022 for one radiation fraction

ROC	Number of patients	Range of travel distance	Average distance traveled for one radiation fraction (SD)	Adjusted range after removing outliers^a^
UKMD^b^	88	4.6–302.4 km	58.3 km (45.8 km)	4.6–206.0 km
KL	640	1.2–862.7 km	42.7 km (33.3 km)	1.2–153.2 km
UKE	1018	1.0–605.5 km	14.0 km (10.8 km)	1.0–51.3 km
UKM	1779	1.4–1873.1 km	37.3 km (30.4 km)	1.4–136.9 km
UKJ	880	0.9–745.1 km	55.5 km (42.9 km)	0.9–186.1 km

*ROC* radiation oncology center, *SD* standard deviation, *UKMD* University Hospital Magdeburg, *KL* Klinikum Landshut, *UKE* University Hospital Hamburg Eppendorf, *UKM* University Hospital Münster, *UKJ* University Hospital Jena

^a^ Cutoffs were determined using the interquartile range (IQR). Data points situated more than 1.5 times the interquartile range above the third quartile (Q3) or below the first quartile (Q1) were considered outliers

^b^ Information from UKMD only available for 2022

All analyses were conducted in R statistical software version 3.2.3 [35].

### Ethical considerations

Before data collection, the protocol for this analysis was approved by the ethics committee of the Bavarian State Medical Association (BLAEK; vote 2022-1216). All data were processed anonymously. Only meta-data were pooled for the final analysis.

## Results

In total, addresses from 4198 breast cancer patients treated between 2018 and 2022 (KL: 617; UKE: 914; UKJ: 870; UKM: 1711; and UKMD: 86 patients) were included in the analysis.

**Fig. 2:**
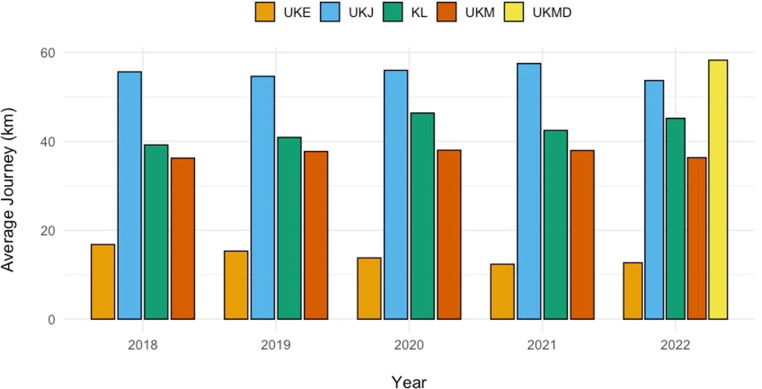
Average travel distance undertaken by breast cancer patients during 2018–2022 for one irradiation fraction along with standard deviation (information for UKMD was only available for 2022). *UKMD* University Hospital Magdeburg, *KL* Klinikum Landshut; *UKE* University Hospital Hamburg Eppendorf, *UKM* University Hospital Münster, *UKJ* University Hospital Jena

The average distances for a round trip were as follows: KL 42.6 km, UKE 14.2 km, UKM 37.3 km, UKJ 55.5 km, and UKMD 58.3 km (Table 1; Fig. 2).

## Discussion

By quantifying travel distances, our study reveals the potential environmental repercussions of RT treatment, particularly the CO_2_ emissions from patients commuting to oncology centers. The primary focus of hypofractionation is to enhance treatment efficiency and improve patient quality of life by reducing the number of hospital visits with the added benefit of reducing the environmental footprint, thus bringing healthcare practices into harmony with ecological sustainability.

### Average distance

In accordance with 2011 census data, approximately 55 million people or 68% of inhabitants live within an urban area and 25 million people or 32% in rural areas. The average trip distance was 18.6 km, amounting to 37.2 km for each round visit. However, the participating ROCs showed a high variability in terms of travel distance. For example, the ROC of the University Hospital Magdeburg is located within an urban area, but the averaged single trip is as long as 25 km. In comparison, the University of Hamburg ROC is also within an urban area, but the averaged single trip is just 7.3 km. One explanation might be the number of alternative treatment institutions within the direct vicinity of the selected ROC. Magdeburg has two ROCs, with the next closest facilities located at significantly greater distances. The mean distance to the three closest ROCs is as much as 45.6 km. This leads to recruitment of patients from rural areas to the urban ROC. Therefore, it appears plausible that the ROC of the Landshut hospital, which is in a central city of a rural area, has an almost identical averaged single trip (21.3 km). In contrast, according to the German Society for Radiation Oncology (DEGRO), there are eight ROCs in Hamburg, and the mean distance to the three closest ROCs of UKE is as small as 6.95 km [36]. This allows shorter travel distances for patients.

### Carbon footprint

Given the average CO_2_ emission of 0.168 kg CO_2_ per km, the CO_2_ emission solely due to transportation is 6.79 kg per visit, which already exceeds the CO_2_ emissions of ROCs directly caused by a complete RT course using a photon therapy device [26, 37, 38].

The average number of fractions for adjuvant RT in breast cancer patients in Germany remains unknown. A survey of 180 German-speaking radiation oncologists stated in 2017 that 151 participants believed conventional fractionation to be the standard of care in Germany [39]. Even 1 year after adoption of hypofractionation as the standard of care in patients after breast-conserving surgery, especially non-academic centers remained on conventional fractionation regimens, as shown by a secondary analysis of the HYPOSIB trial [40]. A more recent international survey found that in 2018 and 2019, 75% of the responding radiation oncologists from high-income countries preferred hypofractionation for node-negative patients after breast-conserving surgery, but only 35% did so for patients with node-positive disease [40].

Based on these uncertainties, but informed by the survey data, we arbitrary calculated with 27 fractions for patients with node-negative disease, including one visit for gaining informed consent and one additional visit for treatment planning as the starting point for calculation of CO_2_ emissions caused by travel of breast cancer patients in Germany. Similarly, 30 visits, including 28 fractions of RT and one additional visit for informed consent and treatment planning, was considered reasonable. Based on an evaluation by the German Federal Office for Radiation Protection, 42,920 patients received radiotherapy for breast cancer in 2016 [41]. Approximately 25% of breast cancer patients are node positive at diagnosis, while around 75% are node negative [42, 43]. This would result in roughly 38,000 node-negative and 12,500 node-positive cases in 2016. Given the assumed average number of round trips for node-positive and node-negative cases, breast cancer patients would have travelled about 1004 km per course for node-negative disease and 1116 km for node-positive disease, emitting 187 and 169 kg CO_2_ per course, respectively. For the whole population, this results in travelled distances of 12,173,200 km for node-positive patients and 38,167,200 km for node-negative cases, with a total distance of 52,340,400 km, resulting in an annual emission of 8793 tons CO_2_.

Giving a conservative assumption for 2023, treating 80% of node-negative patients with moderate hypofractionation (average number of trips = 20.4) and 95% of node-positive patients with conventional fractionation (average number of trips = 29.4) would reduce the total driving distance per patient to 759 km for node-negative and to 1093 km for node-positive patients. For the whole population, this results in 42,727,176 km total distance. Hence, already this conservative adoption of hypofractionation would result in an annual saving of close to 10 million kilometers of driving distance or a reduction of 1615 tons of CO_2_ emissions. In addition, broad adoption of moderate and ultrahypofractionation, such as shown for Wales during the COVID 19 pandemic, would further decrease the CO_2_ emissions indirectly caused by RT [44].

Similarly, consequent adoption of moderate hypofractionation with 15 fractions and a simultaneous integrated boost, as supported by a large body of evidence, would result in 17 round trips per patient, 30,562,600 km of travel distance, and 5135 tons of CO_2_ emissions. That equals an annual saving of 3659 tons CO_2_ emissions in comparison to 2016.

In 2016, it was estimated that in the UK, full adoption of TARGIT-IORT would save 8 million kilometers of travel, 170,000 woman-hours, and 1200 tons of CO_2_ (a forest of 100 hectares) annually [38]. Extrapolating these data for Germany, it is estimated that use of TARGIT-IORT instead of a whole-breast 3‑week course of fractionated radiotherapy for suitable patients would save 10 million kilometer of travel and 1500 tons of CO_2_ (a forest of 125 hectares) per year.

### Correlation to direct emissions

To put the moderate reduction of CO_2_ emissions into relation with the direct emissions of radiotherapy, we further calculated the estimated CO_2_ emissions due to the treatment machines in Germany. In 2016, German ROCs delivered 201,615 courses of RT for malignant diseases. Assuming an average number of fractions of 25 fractions per course, this would result in about 5 million fractions delivered per year. The average energy consumption is estimated to range from 0.144 to 1.6 kWh per fraction, with a mean of 0.872 kWh, plus an idle energy consumption ranging from 4.5 to 5.8 kWh/fraction, mean 5.15 kWh [29]. In total, this equals 6.022 kWh per fraction. The total emission by the treatment machines in 2016 would be roughly 31,620 MWh. In accordance with statista.com, the average CO_2_ emissions in 2016 were 0.448 kg CO_2_/kWh [45]. This would result in CO_2_ emissions of 14,165 tons of CO_2_/year. Hence, the moderate adoption of hypofractionation for breast cancer patients already resulted in more than 10% compensation in CO_2_ emissions caused by the treatment machines for all malignant indications in 2016 (1615 tons saved by reduction in the number of round trips vs. 14,165 tons of total linac emissions). Further adoption could help to compensate for up to 23% in the case of full adoption of moderate hypofractionation or even more in the case of adoption of ultrahypofractionation.

### Incentives

Noteworthily, the German guideline for breast cancer is conservative with regard to hypofractionated RT. Moderately hypofractionated RT was adapted to the German guideline as late as in 2017, and ultrahypofractionation is not recommended in the newest interdisciplinary S3 guideline. Besides adoption of moderate hypofractionation in the guideline, the fee-for-service model in Germany places strong incentives for longer and more intensive treatments. This is well documented for palliative RT for painful bone metastases but also holds true for other indications [46, 47]. Recent surveys, including one in 2017 among breast cancer radiotherapists, indicate financial considerations as a notable barrier, with 19.9% of respondents reluctant to adopt hypofractionated RT due to economic concerns. These findings underscore the influence of financial and procedural incentives on treatment choices. Consequently, it is crucial to advocate for a healthcare model that prioritizes patient-centered outcomes, operational efficiency, and ecological considerations, particularly amidst the challenges posed by climate change and the energy crisis. Nonetheless, the focus on financial aspects should not overshadow the array of broader benefits that hypofractionation brings. These benefits extend beyond direct economic implications, significantly affecting healthcare efficiency and patient wellbeing.

### Benefits

Prioritizing patient-oriented outcomes is paramount, and extensive research has already demonstrated that hypofractionation effectively meets these criteria. The shift towards hypofractionation not only enhances clinical efficacy and environmental conservation but also touches upon several critical yet under-discussed facets of healthcare delivery. It promotes operational efficiency within ROCs by optimizing the use of radiation therapy equipment and reducing the demand on healthcare personnel. This efficiency is crucial in environments where resource constraints are a constant challenge. Moreover, hypofractionation significantly mitigates the physical and emotional burden on patients by shortening the overall treatment timeline, aligning care more closely with patient needs and preferences. In addition to improving the patient experience, fewer trips to the oncology center result in lower fuel costs and reduced CO_2_ emissions, an important consideration in the context of climate change and high energy prices.

### Limitations

Methodologically, there are some limitations that need to be discussed regarding the calculated average travel distances as well as assumptions leading to the CO_2_ emissions. Firstly, a limited number of centers and cases were included into this analysis. Secondly, the average travel distance assumes that all patients drive by car, using the most convenient travel distance as recommended by Google Maps. However, especially in metropolitan areas, patients might arrive as pedestrians or by public transportation, which would reduce the CO_2_ emission. Furthermore, the travel distance calculation was performed for arbitrary times. There might be differences in the travel distance in real live, as the current amount of traffic might influence the timeliness of the shortest route.

While representing real-life data for the selected areas, generalizing them to the whole population of Germany is likely to include some error. This error propagates to the calculation of CO_2_ emissions, which is based on assumptions regarding the average CO_2_ emission per kilometer when driven with a conventional four-wheel car run on fossil fuels. However, the motivation for this analysis was to inform about the magnitude of potential reductions while presenting an exact average travel distance. Additionally, while TARGIT-IORT was not employed in the five participating clinics, it represents an area for future research to further explore its potential CO_2_ savings.

## Conclusion

This study estimates the average travel distance as well as the CO_2_ emissions caused by travel to the ROCs for breast cancer radiotherapy. The estimation is informed by real-life data from 4198 patients from five ROCs in Germany. The data show that broad adoption of hypofractionated regimens has the potential to elicit a significant reduction in CO_2_ emissions. However, the primary consideration remains improving patient care and treatment efficiency.

## Data Availability

The datasets generated and/or analyzed during the current study are not publicly available but are available from the corresponding author upon reasonable request.
